# The Late Dr. D. C. O’Keefe

**Published:** 1871-09

**Authors:** 


### THE LATE DR. D. C. O'KEEFE.

Atlanta Academy of Medicine,
September 22, 1871.

The committee appointed, at a late meeting, to draft suitable resolutions, as a mark of our respect for the memory of the late Dr. D. C. O'Keefe, make the following report:

Since, by a dispensation of Providence, Dr. D. C. O'Keefe, an eminent and valued member of our profession, and of the Atlanta Academy of Medicine, has been removed from our midst by the inevitable hand of death—cut off in the midst of an honorable and useful life—falling, it would seem, ere his work was finished—

*Be it Resolved*, That while we must submit to the decrees of his Creator, we feel that his loss is a calamity to be deeply deplored by us, as individuals, and as a fraternity; but we feel that there are those to whom the loss is truly irreparable, and would, in this sense, tender the bereaved family our deepest sympathies.

*Resolved further*, That his life and character, his influence and example among us, were such as to make it meet that we here preserve among our records, an outline of his history.

Dr. O'Keefe was born in Ireland, in the year 1827. Remaining in his native land till he had nearly reached maturity, he received that classical education, and had engrafted in him those habits of study and thought, that served as a foundation for the culture and scholarly attainments that characterized him in after years. In 1845, he left the land of his birth, and sought, across the seas, a field that offered the honors and emoluments to which a commendable ambition aspired.

Selecting our State as his home, and then choosing ours as his profession, he began its study under the instruction of the Drs. Campbell, of Augusta. Here began a friendship that survived time and absence, and outlived years of engrossing cares and arduous toil. 'Twas but a few months since, on the occasion of the last meeting of the Georgia Medical Association, that Dr. H. F. Campbell, its President, alluded, in terms of tender regard, to the absent friend of by-gone years.

Dr. O'Keefe was well adapted, by his habits, application, and disposition, to the profession of his choice. In no other avocation could some of the best qualities of his mind have been so well brought into requisition. Once espoused, it was pursued with unwearying diligence. His was an intellect of no ordinary type. His success was gratifying to the teachers, and elicited the applause of his classmates. Those of them who knew him best believed him destined to tread that path of honor and renown that leads through a life of usefulness. His deportment was unpretending, and so fired no heart with envy. In later years, remembering his diligence, they marked with pleasure, each successive step in the advance to the fulfillment of their early predictions. His graduating thesis comprised the result of original investigations in a desirable field; and as such, was extensively copied in the medical press throughout the country.

After graduating, in 1849, he began practice at Penfield, Greene county. His first care—prompted not less by gratitude than justice—was to discharge obligations, of necessity incurred, in the acquirement of his education. Removing, shortly, to Greenesboro', he continued a large practice till ill-health forced him to seek recuperation in East Tennessee. After two years' residence at Knoxville, returning health allowed him, in 1859, to locate in Atlanta, and resume the practice of the profession he had relinquished but with regret. Wherever he lived, 'twas his fortune to draw around him friends who watched him with interest till the day of his death.

His course during our late war was an honorable one. He entered the army at its commencement, and continued in the service till its close. He filled many high and important positions in the medical department. Throughout all of our Southern States are many who lend their testimony to his skill and efficiency in those dark hours of our history.

After the close of the war, he returned to Atlanta, and again enjoyed a large and lucrative practice. He loved his profession, and prosecuted it with zeal. In 1865, he was elected to the Chair of Anatomy in the Atlanta Medical College. He was shortly transferred from this to that of the Theory and Practice of Medicine. His lectures were rendered instructive by his fine intellect, and were adorned by the graces of culture and refinement. More scarcely need be said of his course among us. One trait he possessed—that of professional honor—that only a physician can fully appreciate. He regarded this punctiliously—not merely because of its requirement by the code of ethics, but because whatever was inconsistent with right was repugnant to his nature.

More recently he was connected with the government of our city. Here, his prudence and integrity, his energy and enterprise, combined as they were, with urbanity, commanded the encomiums of our citizens. On the subject of general education he was almost an enthusiast. His benevolence saw in this the remedy for many of our ills. Thus would he sap the root of crime, diminish the ills of poverty, even limit the ravages of disease. In the same way he would add beauty and attractions to our homes, and make our State bloom with prosperity. He was author of the measure for the establishment of public schools in our city; and to his perseverance is due, in great part, the system of education now about to be inaugurated under such auspicious circumstances.

While a man of science, he possessed traits that we must admire: brighter were the qualities that endeared him to those who knew him in the walks of private life; one who knew him but casually could not fail to observe the gentleness of his manner, and his unassuming, unaffected demeanor.

This was an index to his character—was a part of the man—and will be always associated with the remembrance of his name. Those who knew him well recognized the sympathy of his nature, and his consideration for all: his heart was ever open to the misfortunes of his fellow-man; his tears were ever ready to start at the tale of distress, or to mingle with those of a friend.

But though we might know Dr. O'Keefe as a companion, or love him as a friend, we would still fall short of the full appreciation of the character in which he shone pre-eminent. 'Tis in his home that the excellence of a good man is most manifest. It can surely be no profanity of the sacredness of domestic relations to admire the qualities that made him the idol of a family. Earth knows no affection stronger than was his; no kindness more self-sacrificing; no tenderness more unceasing. No unkind word passed his lips, and no unkind thought found a home in his heart. His family was his all; and their happiness the one object of his life. To him death could offer no terrors greater than the thought of leaving behind those who must so deeply mourn.

Let us esteem him as a man, and admire him as a physician; but the more should we honor him as embodying so many of the qualities that relieve human nature of its harshness, and rebuke those who hold that the battle of life can be fought but in sordidness.

